# Image Quality and Lesion Detectability of Low-Concentration Iodine Contrast and Low Radiation Hepatic Multiphase CT Using a Deep-Learning-Based Contrast-Boosting Model in Chronic Liver Disease Patients

**DOI:** 10.3390/diagnostics14202308

**Published:** 2024-10-17

**Authors:** Yewon Lim, Jin Sil Kim, Hyo Jeong Lee, Jeong Kyong Lee, Hye Ah Lee, Chulwoo Park

**Affiliations:** 1 Department of Radiology, College of Medicine, Ewha Womans University, Seoul 07985, Republic of Korea; jennyannalim@gmail.com (Y.L.); hjleerad@ewha.ac.kr (H.J.L.); kyongmd@ewha.ac.kr (J.K.L.); 2 Clinical Trial Center, Mokdong Hospital, Ewha Womans University, Seoul 07985, Republic of Korea; khyeah@ewha.ac.kr; 3 Siemens Healthineers Ltd., Seoul 06620, Republic of Korea; chulwoo.park@siemens-healthineers.com

**Keywords:** radiation, multidetector computed tomography, deep learning, image reconstruction, low iodine concentration

## Abstract

Background: This study investigated the image quality and detectability of double low-dose hepatic multiphase CT (DLDCT, which targeted about 30% reductions of both the radiation and iodine concentration) using a vendor-agnostic deep-learning-based contrast-boosting model (DL-CB) compared to those of standard-dose CT (SDCT) using hybrid iterative reconstruction. Methods: The CT images of 73 patients with chronic liver disease who underwent DLDCT between June 2023 and October 2023 and had SDCT were analyzed. Qualitative analysis of the overall image quality, artificial sensation, and liver contour sharpness on the arterial and portal phase, along with the hepatic artery clarity was conducted by two radiologists using a 5-point scale. For quantitative analysis, the image noise, signal-to-noise ratio, and contrast-to-noise ratio were measured. The lesion conspicuity was analyzed using generalized estimating equation analysis. Lesion detection was evaluated using the jackknife free-response receiver operating characteristic figures-of-merit. Results: Compared with SDCT, a significantly lower effective dose (16.4 ± 7.2 mSv vs. 10.4 ± 6.0 mSv, 36.6% reduction) and iodine amount (350 mg iodine/mL vs. 270 mg iodine/mL, 22.9% reduction) were utilized in DLDCT. The mean overall arterial and portal phase image quality scores of DLDCT were significantly higher than SDCT (arterial phase, 4.77 ± 0.45 vs. 4.93 ± 0.24, AUC_VGA_ 0.572 [95% CI, 0.507–0.638]; portal phase, 4.83 ± 0.38 vs. 4.92 ± 0.26, AUC_VGA_ 0.535 [95% CI, 0.469–0.601]). Furthermore, DLDCT showed significantly superior quantitative results for the lesion contrast-to-noise ratio (7.55 ± 4.55 vs. 3.70 ± 2.64, *p* < 0.001) and lesion detectability (0.97 vs. 0.86, *p* = 0.003). Conclusions: In patients with chronic liver disease, DLDCT using DL-CB can provide acceptable image quality without impairing the detection and evaluation of hepatic focal lesions compared to SDCT.

## 1. Introduction

Hepatocellular carcinoma (HCC) is the third leading cause of cancer-related mortality [1,2]. Hepatic multiphase computed tomography (CT) is the main imaging modality used for evaluating focal liver lesions in patients with underlying chronic liver disease or liver cirrhosis [3,4]. Continuous and intense surveillance to detect early recurrence is crucial for such individuals. These patients usually undergo repetitive hepatic multiphase CT scans during the follow-up period, resulting in double the radiation exposure compared to a single-phase abdominopelvic CT scan, and requiring intravenous injection of iodinated contrast media.

Exposure to iodinated contrast media may lead to immediate hypersensitivity reactions and acute kidney injury [5,6,7]. Depending on the osmolality and viscosity of the contrast medium, urine viscosity in the renal tubules may increase after the contrast injection. This, in turn, may increase the risk of kidney injury due to the increased pressure in the renal tubules and parenchyma [8,9,10,11]. If the iodine concentration in the contrast medium decreases, the osmolality and viscosity also decrease, thus reducing the possibility of contrast media complications. To detect arterial enhancement and determine washout patterns, hepatic multiphase CT scans commonly use a contrast medium with an iodine concentration of 350 mg iodine/mL or more, which is higher than that of typical single-phase abdominopelvic CT scans [12,13]. Therefore, determining the usage of low-concentration iodine contrast media in hepatic multiphase CT may contribute to decreasing complication rates.

Reducing iodine contrast may come at the expense of compromising the diagnostic efficacy; therefore, ensuring both adequate image quality and iodine dosage is essential. Recently, various techniques have been introduced to reduce the radiation doses, with deep-learning-based algorithms emerging as a promising approach [14,15,16,17]. Similarly, recent studies suggest various techniques to maintain acceptable image quality while reducing the iodine contrast dose, ranging from low tube voltage and iterative reconstruction to deep-learning-based reconstruction models [18,19,20,21]. Various deep-learning models, particularly those focusing on contrast-boosting algorithms, have showed the possibility of reducing the radiation dose [14,19] or reducing the iodine contrast burden by lowering the volume of the contrast medium [19,21]. A previous study reported the possibility of reducing both the radiation dose and contrast volume by 19.8% and 27%, respectively, by using a deep-learning-based contrast-augmenting algorithm [19]. Another recent study using deep-learning-based contrast-boosting techniques showed acceptable lesion conspicuity in single-phase abdominal CT scans conducted with low-concentration iodine contrast (240 mg iodine/mL). However, the study population was limited to pediatric patients with underlying malignancy [22]. Whether hepatic multiphase CT scanning using a contrast medium with a lower iodine concentration, which directly affects the osmolality and viscosity, shows acceptable diagnostic performance in hepatic evaluation for adults, remains elusive.

Therefore, this study aimed to investigate the image quality and detectability of double low-dose hepatic multiphase CT (DLDCT, which targeted about 30% reductions of both radiation and iodine concentration) using a vendor-agnostic deep-learning-based contrast-boosting (DL-CB) algorithm compared to those of standard-dose CT (SDCT) using hybrid iterative reconstruction.

## 2. Materials and Methods

### 2.1. Patient Population

This retrospective study was approved by the institutional review board of our institution. 

**Figure 1:**
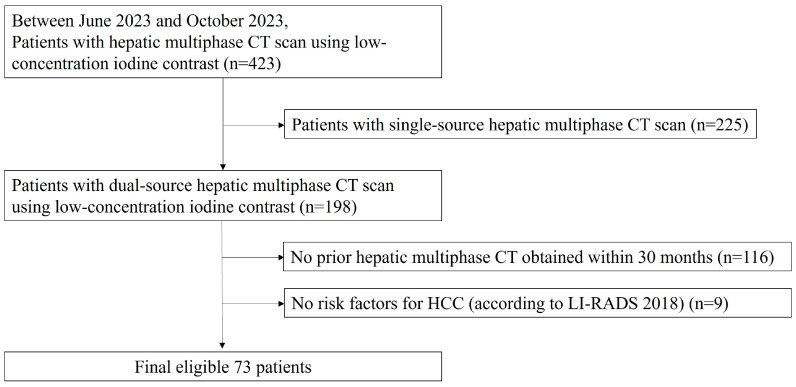
Participant flowchart.

Between June 2023 and October 2023, 423 patients underwent hepatic multiphase CT scans using low-concentration iodine contrast (270 mg iodine/mL). Of these, 18 patients underwent hepatic multiphase CT scans using low-concentration iodine contrast (270 mg iodine/mL) more than once. The patients who underwent single-source hepatic multiphase CT scans were excluded (*n* = 225). Among them, the patients without a prior hepatic multiphase CT using 350 mg iodine/mL as contrast, obtained within the past 30 months (*n* = 116) were excluded. The patients without an underlying disease that is considered a risk factor for developing HCC according to the Liver Imaging Reporting and Data System (LI-RADS), version 2018 [3], (*n* = 9) were also excluded. Finally, 73 eligible patients were included in this study (Figure 1).

### 2.2. CT Scanning Protocol and Image Reconstruction

All CT scans were conducted once, using a 192-detector row CT scanner (SOMATOM Force; Siemens Healthineers, Forchheim, Germany) with automated tube current modulation (CareDose4D, Siemens Healthineers). We employed the dual-source mode, wherein “tube A” administered two-thirds (66.7%) of the overall radiation dosage, while “tube B” administered one-third (33.3%) of the total radiation dosage [23]. The CT protocol included unenhanced, hepatic arterial, portal venous, and delayed phases. All phases used the same imaging parameters: 128 × 0.6 mm collimation, 0.6 pitch, 3 mm reconstruction interval, 90 (less than 60 kg) or 100 kVp, and 169–260 mAs. The scan range for the unenhanced, hepatic arterial, and delayed scans covered the area from the lower chest to the inferior liver pole, while the portal venous phase covered the area regions from the lower chest to the pelvic cavity. The unenhanced scans were conducted first. Thereafter, the contrast medium (Iohexol 270 mg iodine/mL [IOBRIX 270, Accuzen]; 463 mg I/kg based on 70 kg) was intravenously injected via an automatic power injector at the rate of 3 mL/s. The administered contrast volume was 110 mL for patients weighing 60 kg or less, and 120 mL for those weighing more than 60 kg. The hepatic arterial, portal venous, and delayed phases were conducted at the following time points: 12 s after reaching a trigger threshold of 100 HU at the abdominal aorta, 80–90 s after contrast administration, and 180 s after contrast administration. The dose-length product was automatically calculated. The effective dose of CT was calculated using the method derived from the European Working Group and the organ weighting factor (k = 0.0151 mSv·mGy^−1^·cm^−1^) [24]. The DLDCT was reconstructed using a vendor-agnostic deep-learning-based contrast-boosting model (DL-CB, ClariCT.ACE, ClariPI).

The DL-CB model used in this study is a commercially available vendor-neutral image reconstruction technique used in previous studies [19,21]. It was developed as a contrast-boosting algorithm for low-dose contrast-enhanced CT scans based on the U-net architecture. The model consists of the following two stages: the denoising stage and the contrast-boosting stage. Each stage incorporates an encoder module and decoder module using a concatenated skip connection [25]. Further details of the DL-CB model are presented in Appendix A.

The SDCTs were performed with two different multi-detector CT scanners (SOMATOM Force and SOMATOM Definition flash; Siemens Healthineers, Forchheim, Germany), with automated tube current modulation (CareDose4D, Siemens Healthineers) and automated tube voltage control (CARE kV, Siemens Healthineers). All phases were obtained using the same bolus triggering technique as per the DLDCT, using either 110 mL (for patients weighing 60 kg or less) or 120 mL (for patients weighing more than 60 kg) of contrast (Iohexol or Iobitridol 350 mg iodine/mL; 600 mg I/kg based on 70 kg). Dual-energy (DE) scans were used for the arterial phase. The imaging parameters were as follows: for SOMATOM Force, 128 × 0.6 mm collimation, 0.6 pitch, 3 mm reconstruction interval, and two different tube voltages (80 kV and tin filtered 150 kV, reference tube currents 325/163 mAs). The DE scans were reconstructed into a blended image with a mixed ratio of 0.6 (60% 80 kV and 40% Sn150 kV); for SOMATOM Flash, 32 × 0.6 mm collimation, 0.6 pitch, 3 mm reconstruction interval, and two different tube voltages (100 kV and tin filtered 140 kV (Sn140 kV), reference tube currents 219/170 mAs). The DE scans were reconstructed into a blended image with a mixed ratio of 0.5 (50% 100 kV and 50% Sn140 kV). We used blended images for the arterial phase evaluation. Single-energy (SE) scans were used for the portal and delayed phases. The imaging parameters were as follows: for SOMATOM Force, 192 × 0.6 mm collimation, 0.6 pitch, 3 mm reconstruction interval, the reference tube voltage 120 kV, and the reference tube current 150 mAs; for SOMATOM Flash, 128 × 0.6 mm collimation, 0.8 pitch, 3 mm reconstruction interval, the reference tube voltage 120 kV, and the reference tube current 150–180 mAs. The SDCT was reconstructed using a hybrid iterative reconstruction technique from ADMIRE 3. 

### 2.3. Image Analysis

All 73 pairs of the arterial phase and portal phase CT examinations (SDCTs and DLDCTs) were evaluated by two radiologists (J.S.K. with 12 years of experience and H.J.L. with 6 years of experience in abdominal CT interpretation) independently. Both investigators were blinded to which protocol each CT scan was taken with. All reviews were performed using our clinical picture-archiving and communication system (PACS, INFINITT Healthcare, Seoul, Republic of Korea) and allowed to optimally adjust the window width and level. The overall image quality and artificial sensation on the arterial and portal phases were evaluated using a 5-point scale (the higher the score, the better image quality) as follows: 1, very poor (non-diagnostic quality and reexamination needed); 2, suboptimal (unsatisfactory quality but reexamination not needed); 3, average; 4, better than average; and 5, excellent image quality without related issues of concern [14,23]. The hepatic artery clarity of the arterial phase was also evaluated with a 5-point scale as follows: 1, very poor; 2, suboptimal; 3, average; 4, better than average; 5, excellent (the second branch of the hepatic artery is well detected).

The quantitative analysis including noise, signal-to-noise ratio (SNR), and contrast-to-noise ratio (CNR) was performed by a single radiologist (Y.L. with 2 years of experience in abdominal radiology). For the noise and SNR evaluations, a circular region of interest (ROI) measuring about 1–3 cm^2^ was drawn at the homogenous area of each liver section (right anterior, right posterior, left medial, and left lateral sections) at the portal vein level on the arterial phase transverse image of the SDCT and DLDCT scans. The noise of the liver parenchyma was determined as the mean SD of the four measurements. The SNR was calculated based on the mean HU of the four measurements according to the following equation: SNR = mean HU of the liver (arterial)/noise. For the CNR evaluation, arterial enhancing lesions that had the same detectability on both the DLDCT and SDCT scans were analyzed, as the degree of arterial enhancement in focal lesions is an important factor in reducing the iodine dosage. For each lesion, the ROI was manually drawn to encompass the focal lesion as fully as possible. Each focal lesion ROI was measured three times, and the average value was used. The CNR was calculated using the following equation: CNR = (mean HU of focal arterial enhancing lesion − mean HU of the liver (arterial))/noise.

The focal lesion evaluation was independently performed by the two independent board-certified radiologists (J.S.K. with 12 years of experience and H.J.L. with 6 years of experience in abdominal CT interpretation) who also performed the qualitative analysis. One senior radiologist (J.K.L. with more than 20 years of experience) initially marked the lesions detected in the CTs. Correlation with all available clinical data, including the electronic medical records, surgical or biopsy pathology results, and findings from the index and comparison imaging studies (CT, MR, and PET/CT), was conducted for the reference standard. Wedge-shaped typical arterioportal shunts were not included in this study. The two independent radiologists then evaluated the lesion conspicuity of the marked lesions using a 5-point scale (score of 1, not distinct; 2, barely distinct; 3, moderately distinct; 4, fairly distinct; 5, definitely distinct). The lesions graded 3–5 were counted as detected lesions, while those graded 1–2 were counted as non-detected lesions. Additionally, each lesion was categorized according to LI-RADS criteria [3]. The lesions reported as LR-4 or LR-5 were regarded as representing HCC. 

### 2.4. Statistical Analysis

Summary statistics of the patients were presented as mean and standard deviation with ranges for continuous data, and the number of subjects along with percentages for categorical data. Continuous variables including dose parameters and quantitative analysis results were analyzed with paired *t*-test using commercially available statistical software (IBM SPSS Statistics for Windows, v. 29.0; IBM, Armonk, NY, USA; or MedCalc, v. 19.2.1; MedCalc, Marikerke, Belgium). Two-tailed *p*-values < 0.05 were considered statistically significant.

For the comparison of the qualitative and focal lesion analysis results including the overall image quality, artificial sensation, and hepatic artery clarity between the SDCT and DLDCT, visual grading characteristics (VGC) analysis was conducted using an open-source software (JROCFIT, https://www.rad.jhmi.edu/jeng/javarad/roc/JROCFITi.html, accessed on 1 March 2024). A VGC curve was generated in a manner similar to receiver operating characteristic (ROC) curves using the distribution of ratings for SDCT and DLDCT. The average area under the VGC curve (AUC_VGC_) provided a statistically valid nonparametric measure of differences across each analyzed parameter. An AUC_VGC_ greater than 0.5 indicated a statistically significant difference between the two CTs [26]. The interobserver agreement was assessed using the weighted kappa (k). Agreement was considered as follows: slight (0.01–0.20), fair (0.21–0.40), moderate (0.41–0.60), substantial (0.61–0.80), and almost perfect (0.81–1.00) [27]. The lesion conspicuity and lesion detection were analyzed using generalized estimating equation (GEE) analysis because of the presence of multiple lesions in a patient. It was estimated with point estimates and 95% confidence intervals (95% CI).

## 3. Results

A total of 73 patients (57 men and 16 women) were included in this study. The mean body weight was 68.9 ± 14.9 kg (range: 41.7–121.4 kg) and the mean body mass index (BMI) was 25.0 ± 4.4 kg/m^2^ (range: 14.9–38.2 kg/m^2^). The underlying chronic liver disease demographics are in Table 1. The time interval between DLDCT and SDCT was 9.2 ± 8.2 months. Four patients had more than a 10% difference of body weight between the two CT scans (one patient gained 13.8% of body weight, while three patients lost an average of 17.5% of body weight [18.5%, 19.8%, and 14.2%]). 

Compared with the SDCT, the mean DLP of the DLDCT was lower by approximately 36.2% (690.7 ± 398.6 [248–2589] mGy × cm vs. 1083.0 ± 477.2 [456–3392] mGy × cm, *p* < 0.001). The mean effective dose of DLDCT was also significantly lower by approximately 36.6% than that of SDCT (10.4 ± 6.0 [3.7–43.2] mSV vs. 16.4 ± 7.2 [6.9–51.2] mSV, *p* < 0.001) (Table 1). The amount of iodine in the contrast was also significantly lower by approximately 22.9% between the DLDCT and SDCT (270 mg iodine/mL vs. 350 mg iodine/mL).

### 3.1. Qualitative Analysis

The overall image quality and hepatic artery clarity of the DLDCT arterial phase were significantly higher than those of the SDCT (overall image quality AUC_VGA_, 0.572 [95% CI, 0.507–0.638]; hepatic artery clarity AUC_VGA_, 0.587 [0.521–0.652]). However, the artificial sensation of the DLDCT on the arterial phase was significantly lower than that of the SDCT (AUC_VGA_, 0.424 [0.359–0.490]) (Table 2). The inter-reader agreement between the two reviewers was fair or moderate for the artificial sensation (k = 0.281), hepatic artery clarity (k = 0.364), and overall image quality (k = 0.589).

### 3.2. Quantitative Analysis

The noise was significantly lower in DLDCT than that of SDCT (9.77 ± 5.51 vs. 13.81 ± 9.99, *p* = 0.003). Both the SNR of the liver and CNR of the focal lesion were significantly higher for DLDCT than SDCT (SNR, 8.56 ± 1.88 vs. 5.92 ± 1.99, *p* < 0.001; CNR, 7.55 ± 4.55 vs. 3.70 ± 2.64, *p* < 0.001). A total of 24 arterial enhancing lesions in both CTs were evaluated for CNR, including 1 HCC, 3 hemangiomas, and 20 nodular AP shunts.

### 3.3. Focal Lesion Evaluation

**Figure 2:**
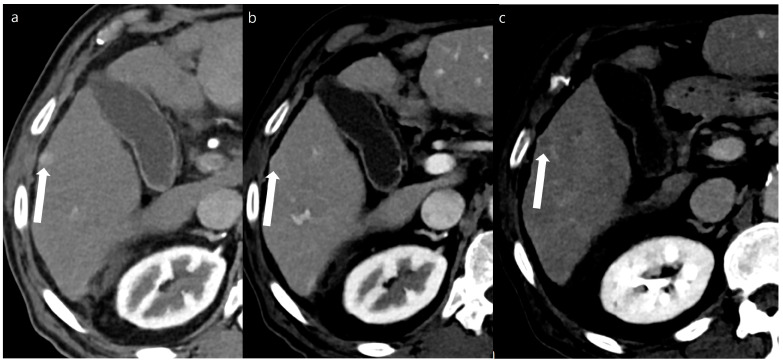
A 65-year-old man with a 0.8 cm sized hepatocellular carcinoma at segment 5 of the liver (arrows). Arterial phase (**a**), portal phase (**b**), and delayed phase (**c**) images of a double low-dose CT using a deep-learning-based contrast-boosting model show a well-enhancing nodule on the arterial phase (**a**), with washout on the portal phase (**b**) and the delayed phase (**c**).

**Figure 3:**
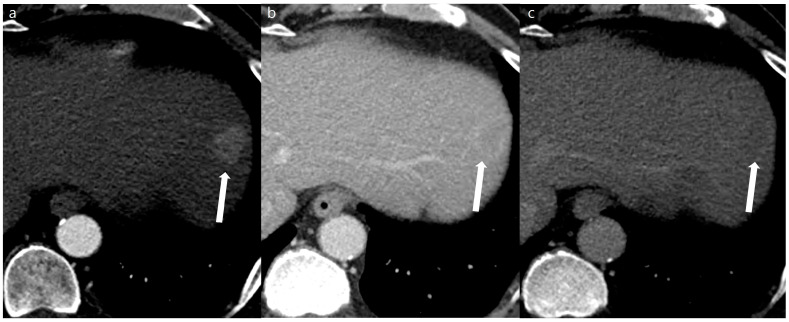
A 53-year-old man with a 2 cm sized hepatocellular carcinoma at segment 2 of the liver (arrows). Arterial phase (**a**), portal phase (**b**), and delayed phase (**c**) images of a standard-dose CT using hybrid iterative reconstruction show a well-enhancing nodule in the arterial phase (**a**), with washout on the portal phase (**b**) and the delayed phase (**c**).

A total of 108 focal liver lesions were determined in 48 patients (101 lesions in the SDCT [*n* = 45] and 98 lesions in the DLDCT [*n* = 44]). Of these, 91 lesions were identified as the same lesions in both the SDCT and DLDCT without interval change. These included 3 HCCs, 1 abscess, 9 hemangiomas, 6 dysplastic nodules (1 HCC on DLDCT), 19 nodular AP shunts, and 53 cysts or small focal low densities. One of the six dysplastic nodules detected in the SDCT showed arterial enhancement in the follow-up DLDCT taken 3 months later, and therefore was characterized as HCC. Eight out of ten lesions that were only visible on the SDCT were HCCs, which required RF ablation or TACE (trans-arterial chemoembolization) treatment. Two others were nodular AP shunts which were not visible on the follow-up DLDCT. Seven lesions that were only visible in the DLDCT included one HCC and six nodular AP shunts. In total, 11 and 5 HCCs were identified from the SDCT and DLDCT, respectively (Figure 2 and Figure 3). Additionally, the mean lesion size was 10.53 ± 11.9 mm (SDCT, 10.7 ± 12.3 mm vs. DLDCT, 9.89 ± 11.4 mm, *p* = 0.625). 

**Figure 4:**
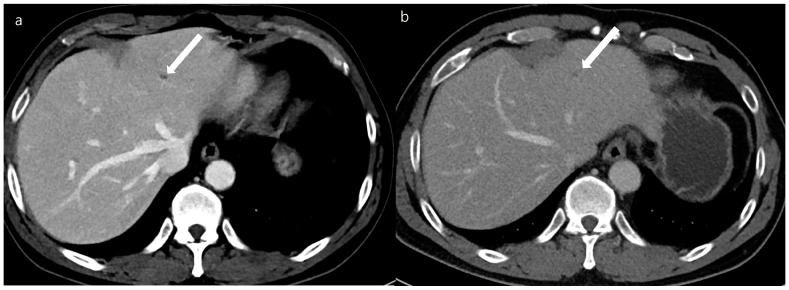
A 54-year-old man with a 0.4 cm sized small focal low density at segment 2 of the liver (arrows). A double low-dose CT using a deep-learning-based contrast-boosting model (**a**) shows better conspicuity (Reviewer 1, 4; Reviewer 2, 5) of the focal lesion (arrows) than standard-dose CT using hybrid iterative reconstruction (Reviewer 1, 2; Reviewer 2, 1) (**b**) at portal phase images (time interval: 11 months). Two reviewers evaluated the double low-dose CT using a deep-learning-based contrast-boosting model and standard-dose CT using hybrid iterative reconstruction. The results demonstrated no difference in overall image quality.

**Figure 5:**
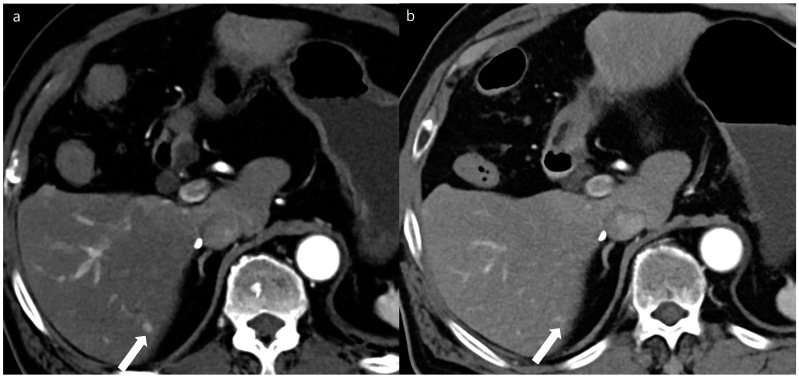
An 82-year-old man with a 0.5 cm sized enhancing nodule at segment 6 of the liver (arrows). A double low-dose CT using a deep-learning-based contrast-boosting model (**a**) shows better conspicuity (Reviewer 1, 5; Reviewer 2, 4) of the focal lesion (arrows) than standard-dose CT using hybrid iterative reconstruction (Reviewer 1, 5; Reviewer 2, 3) (**b**) at arterial phase images (time interval: 4 months). Two reviewers evaluated the double low-dose CT using deep-learning-based contrast-boosting model and standard-dose CT using hybrid iterative reconstruction. The results demonstrated no difference in overall image quality.

Lesion detectability and conspicuity were significantly higher on the DLDCT than on SDCT (lesion detectability, 0.97 [95% CI 0.91–0.99] vs. 0.86 [95% CI 0.79–0.91], *p* = 0.003; lesion conspicuity, 4.43 [95% CI 4.29–4.58] vs. 3.83 [95% CI, 3.63–4.02], *p* < 0.001) (Table 3 and Figure 4 and Figure 5). As for the per-lesion sensitivity of HCC diagnosis (LR-4 or LR-5) by the two reviewers, the DLDCT revealed a sensitivity of 80.0% (8/10 [95% CI, 44.4–97.5]), while the SDCT showed a sensitivity of 50.0% (11/22 [95% CI, 28.2–71.8]). The difference was 30.0% (95% CI, −6.5% to 54%) which was not statistically significant (*p* = 0.115).

## 4. Discussion

The mean overall arterial and portal phase image-quality scores of the DLDCT were acceptable compared to those of the SDCT (arterial phase, overall image quality AUC_VGA_, 0.572 [95% CI, 0.507–0.638]; portal phase, overall image quality AUC_VGA_, 0.535 [95% CI, 0.469–0.601]), although significantly lower effective dose (10.4 ± 6.0 [3.7–43.2] mSV vs. 16.4 ± 7.2 [6.9–51.2] mSV, *p* < 0.001) and iodine amount (270 mg iodine/mL vs. 350 mg iodine/mL) were utilized for the DLDCT. The mean reduction of the radiation and iodine dosage was 36.6% (16.4 mSv vs. 10.4 mSv) and 22.9% (350 mg iodine/mL vs. 270 mg iodine/mL), respectively. In addition to the lower radiation dose and iodine concentration, the lesion detectability and quantitative results of liver lesions including contrast-to-noise ratio in the DLDCT were also improved compared to those of the SDCT (lesion detectability, 0.97 [95% CI 0.91–0.99] vs. 0.86 [95% CI 0.79–0.91], *p* = 0.003; CNR, 7.55 ± 4.55 vs. 3.70 ± 2.64, *p* < 0.001). 

Our findings are consistent with the results from previous studies. Previous studies using deep-learning-based reconstruction models have shown non-inferior image quality and diagnostic performance in evaluating focal liver lesions, while reducing up to 66.7% of the radiation dose [15,16,17,18]. A prior randomized prospective study assessing focal liver lesions in patients with a high risk of HCC suggested the possibility of reducing the radiation and contrast dose by 19.8% and 27%, respectively, while maintaining diagnostic performance by using a deep-learning-based iodine contrast-augmenting algorithm [19]. While the preceding study has explored the utilization of a low-volume option, our study employed a low-concentration iodine option to effectively reduce contrast burden, along with reducing the radiation dose. Iodine concentration influences factors such as osmolality and viscosity, which are more directly linked to the risk of nephrotoxicity than the overall iodine amount [6,10,11]. Our study demonstrated that the overall image quality of both the arterial and portal phases in DLDCT was comparable to SDCT in lower iodine concentration settings. These findings not only confirm the results of previous studies, but also suggest that DLDCT may be employed for liver evaluation in chronic liver disease patients, offering the advantage of further reducing iodine contrast-related side effects. Specifically, DLDCT reduces the radiation dose and iodine concentration by approximately 30% and 20%, respectively, without compromising the image quality or the evaluation of focal hepatic lesions. Since these patients typically undergo repetitive CT scans, the application of DLDCT to reduce the radiation and iodine burden will be clinically beneficial.

In the qualitative analysis, the overall image quality was higher for the DLDCT compared to the SDCT, but this difference was more significant in the arterial phase images than the portal phase images (arterial phase, 4.77 ± 0.45 vs. 4.93 ± 0.24, AUC_VGA_ 0.572 [95% CI, 0.507–0.638]; portal phase, 4.83 ± 0.38 vs. 4.92 ± 0.26, AUC_VGA_ 0.535 [95% CI, 0.469–0.601]). The difference between the DLDCT and SDCT in the portal phase image was not statistically significant (The 95% CI crosses 0.5). Considering that contrast-boosting effects are more significant in arterial phase images than portal phase images, which are already in an enhanced status, may explain why overall image quality improvement is more evident in the arterial phase images but not as significant in the portal phase images in our study. The hepatic artery clarity showed higher image scores in the DLDCT with DL-CB than the SDCT, which can also be attributed to the contrast-boosting algorithm. Conversely, artificial sensation was lower in the arterial phases of the DLDCT than the SDCT but higher in the portal phases, which may be due to contrast enhancement compensation. Previous studies demonstrating comparable overall image quality in settings with reduced radiation and iodine contrast dose employed contrast-boosting algorithms, which aligns with our findings [19,21]. These results altogether suggest that the augmentation of contrast enhancement through DL-CB may contribute as a major factor. 

Our study showed detectability (0.97 [95% CI 0.91–0.99] vs. 0.86 [95% CI 0.79–0.91) and conspicuity (4.43 [95% CI 4.29–4.58] vs. 3.83 [95% CI, 3.63–4.02], *p* < 0.001) on DLDCT using DL-CB for focal liver lesions were not lower than those on SDCT, including malignant lesions. In the quantitative analysis, the DLDCT showed significantly less image noise (9.77 ± 5.51 vs. 13.81 ± 9.99, *p* = 0.003) and higher SNR and CNR than the SDCT (SNR, 8.56 ± 1.88 vs. 5.92 ± 1.99, *p* < 0.001; CNR, 7.55 ± 4.55 vs. 3.70 ± 2.64, *p* < 0.001). A recent single-center retrospective study including 35 pediatric patients (mean age: 9.2 years [1–17 years]) with underlying malignancy showed comparable lesion conspicuity in single-phase abdominal CT scans using a low-concentration iodine contrast (240 mg iodine/mL) and deep-learning-based contrast-boosting techniques [22]. Although the study population demographics and CT protocol differ, the results align with our findings. Our study focuses on chronic liver disease patients undergoing hepatic multiphase CT, which supports the utilization of DLDCT in real clinical practice for follow-up or surveillance of focal hepatic lesions in patients with the risk of developing HCC. These patients undergo repetitive follow-up CT scans, thereby having a greater clinical importance for reducing the radiation and iodine burden. In particular, by reducing the iodine concentration in the contrast, the burden for patients with low renal function may be alleviated, thereby enabling them to undergo consecutive CT scans during follow-up. Additionally, since hepatic cellular carcinoma requires the detection of subtle arterial enhancement, a more delicate approach for chronic liver disease patients may be necessary. The application of DLDCT may be expanded to general patients regarding the benefits of reducing radiation and contrast use. However, since our study only included chronic liver disease patients, further evaluation would be necessary to assess generalizability.

Our study has several limitations. First, we conducted a retrospective study serially comparing a DLDCT with a prior SDCT of the same patient. Due to the time interval between both CT scans, there is a possibility that the lesion itself may have changed over time, thus contributing to the differences observed between the DLDCT and SDCT images, and thereby, biasing results. Previous studies that used deep-learning-based models to lower the radiation and iodine doses were conducted prospectively, comparing CT scans of different patients. Our study has the advantage of comparing the same lesions in the same patient, which may reduce inter-patient variability compared to previous studies analyzing different lesions in different patients. However, it should be noted that results may be prone to the possibility of interference due to temporal differences. Considering temporal differences, we interpreted the study results in a conservative manner. Even though the DLDCT results were significantly superior relative to the SDCT, we tended to interpret the results to be non-inferior. Perspective studies designed to be free of temporal bias may be warranted to assess the superiority or non-inferiority of DLDCT more accurately. Furthermore, lesions that were the same in both DLDCT and SDCT showed clearer margins in the DLDCT images, which may support the possibility of lowering radiation and iodine concentration with DL-CB. Second, only a single vendor (Siemens Healthineers) was tested in this study. The limit to a single vendor may affect the generalizability of the results, since the DL-CB used in our study is a vendor-agnostic deep-learning model. Additional testing with different vendors and CT protocols will be necessary to evaluate the applicability to other CT systems.

In conclusion, the low-concentration iodine contrast (270mg iodine/mL) and low radiation (reduction of 30% radiation dose) hepatic multiphase CT with a deep-learning-based contrast-boosting model (DL-CB) provided acceptable image quality without impeding the detection and evaluation of hepatic focal lesions compared to the standard-dose CT in patients with chronic liver disease. While this highlights the potential of deep-learning-based reconstruction in significantly reducing the iodine concentration in contrast media and the radiation exposure, future prospective studies with various vendors would be needed to ensure further application.

## Figures and Tables

**Table 1:** Study population demography (*n* = 73).

**Parameter**	**Value**
**Sex (men:women), *n***	57:16
**Age, years**	
Men	62.0 ± 12.6 (34–87) ^†^
Women	64.2 ± 11.3 (46–80) ^†^
**Underlying disease, % (*n*/*N*)**	
Chronic hepatitis B	46.6 (34/73)
Chronic hepatitis C	1.4 (1/73)
Alcoholic liver disease	20.5 (15/73)
Cryptogenic	31.5 (23/73)
**Laboratory findings**	
Albumin, g/dL	4.2 ± 0.6 (2.5–5.1) ^†^
Total bilirubin, mg/dL	1.2 ± 1.2 (0.2–6.8) ^†^
INR	1.2 ± 0.5 (0.9–4.3) ^†^
Platelet count, ×10^3^/mm^3^	153.9 ± 81.7 (33–520) ^†^
AFP, ng/mL	37.0 ± 194.1 (1.4–1431) ^†^
**Body weight, kg**	68.9 ± 14.9 (41.7–121.4) ^†^
**Mean body mass index, kg/m^2^**	25.0 ± 4.4 (14.9–38.2) ^†^
**Mean time interval, months**	9.2 ± 8.2 (0–30) ^†^
**DLP, mGycm**	
Standard dose	1083.0 ± 477.2 (456–3392) ^†^
Dual low dose	690.7 ± 398.6 (248–2589) ^†^
**Effective dose, mSv**	
Standard dose	16.4 ± 7.2 (6.9–51.2) ^†^
Dual low dose	10.4 ± 6.0 (3.7–43.2) ^†^

Note: ^†^ Data are represented as mean and standard deviation. Minimum and maximum values are in parenthesis with hyphens. ‘*n*’ indicates the number of people in that parameter, and ‘*N*’ indicates the total number of enrolled patients. The unit of each parameter is noted after the comma of each criterion. Abbreviations: INR, international normalized ratio; AFP, alpha-fetoprotein; DLP, dose length product.

**Table 2:** Qualitative image-quality analysis between standard-dose CT and double low-dose CT using a deep-learning-based contrast-boosting algorithm.

	SDCT	DLDCT	AUC_VGA_ (95% CI)
**Arterial phase**			
Overall image quality	4.77 ± 0.45	4.93 ± 0.24	0.572 (0.507–0.638)
Artificial sensation	4.93 ± 0.25	4.77 ± 0.33	0.424 (0.359–0.490)
Hepatic artery clarity	4.74 ± 0.42	4.95 ± 0.19	0.587 (0.521–0.652)
**Portal phase**			
Overall image quality	4.83 ± 0.38	4.92 ± 0.26	0.535 (0.469–0.601)
Artificial sensation	4.95 ± 0.19	4.47 ± 0.39	0.286 (0.226–0.346)

Note: Data are mean and standard deviation. Abbreviations: SDCT, standard-dose CT; DLDCT, double low-dose CT.

**Table 3:** Comparison of lesion detectability and lesion conspicuity between standard-dose CT and double low-dose CT using a deep-learning-based contrast-boosting algorithm.

	SDCT	DLDCT	*p*-Value
Detectability	0.86 (95% CI 0.79–0.91)	0.97 (95% CI 0.91–0.99)	0.003
Lesion conspicuity	3.83 (95% CI 3.63–4.02)	4.43 (95% CI 4.29–4.58)	<0.001

Abbreviation: SDCT, standard dose CT; DLDCT, double low-dose CT.

## Data Availability

The raw data supporting the conclusions of this article will be made available by the authors on request.

## Appendix A. Architecture of Deep-Learning-Based Contrast-Boosting Reconstruction Model

The DL-CB model (ClariCT.ACE, ClariPI) is a contrast-boosting algorithm developed for low-dose contrast-enhanced CT scans based on the U-net architecture. The architecture consists of the following two stages: a denoising stage and contrast-boosting stage. Each stage in the U-net architecture incorporates an encoder module and decoder module using a concatenated skip connection [19,25]. The encoder module includes a convolutional layer with a kernel size of 3 × 3, followed by a rectified linear unit activation and a 2 × 2 max-pooling layer. The decoder module is structured similarly but substitutes the max-pooling layer with a 2 × 2 up-sampling layer that uses nearest-neighbor interpolation. Finally, a 1 × 1 convolutional layer is applied in the last layer to reduce the dimensions of the features.

In the denoising stage, a noise component is extracted for a given contrast-enhanced CT image, which is then subtracted from the input image to obtain the denoised image. In our study, this was a pre-trained network for image denoising that was developed and validated previously (ClariCT.AI; ClariPi). Following this, the contrast-boosting stage extracts the iodine component image from the denoised image. This model was trained to accept a synthetic low-contrast contrast-enhanced CT image and to predict the weight-adjusted iodine component image. The final contrast-augmented CT image is obtained by multiplying the predicted iodine component image with a user-defined boosting strength and adding back to the input image.

The DL-CB model was trained using a dual-energy CT-derived training dataset. Synthetic low-contrast-dose contrast-enhanced CT images of varying degrees were regenerated by combining the virtual non-contrast (VNC) image with a weight-adjusted iodine component image of the dual-energy CT with weight ranges from 0.5 to 1.5.
